# Perceptions of sugar-sweetened beverages among adolescents in North Carolina

**DOI:** 10.3389/fpubh.2022.943295

**Published:** 2022-09-29

**Authors:** Lindsey Haynes-Maslow, Sarah Ray, Kristen Giombi

**Affiliations:** ^1^Department of Agricultural and Human Sciences, North Carolina State University, Raleigh, NC, United States; ^2^Center for Communication Science, RTI International, Atlanta, GA, United States; ^3^Health Economics Program, RTI International, Research Triangle Park, NC, United States

**Keywords:** adolescents, sugar-sweetened beverage, marketing, low-income, perceptions

## Abstract

**Introduction:**

Sugar-sweetened beverage (SSB) consumption among adolescents contributes to diet-related chronic disease including obesity, type 2 diabetes, and poor oral health.

**Objective:**

To better understand adolescents' perceptions, attitudes, and consumption behaviors around SSBs by conducting virtual workshop discussions with adolescents in NC.

**Materials and methods:**

Adolescents ages 11–17 in communities with a high proportion of Supplemental Nutrition Assistance Program (SNAP) eligible households were selected to participate in a series of virtual group workshops during summer 2021. A semi-structured discussion guide was used by a workshop facilitator. Workshop discussions centered around general health perceptions, SSB perceptions, and consumption behaviors. A thematic analysis was used to summarize knowledge, beliefs, attitudes, and perceptions around SSBs.

**Results:**

Approximately 36 adolescents participated across four group workshops. Parents and caregivers influenced adolescents most when it came to making beverage choices. Positive SSB perceptions included liking the taste and the association with special times and social events. Negative opinions focused on associated health risks (diet-related chronic disease and poor oral health). Some adolescents acknowledged SSBs were not healthy but suggested they could be consumed occasionally. Very few participants mentioned any benefits from SSBs; those that mentioned benefits stated they provided energy, replaced electrolytes, and tasted good.

**Conclusion:**

Findings provide several key insights that can contribute to the development of messages aimed at curbing SSB consumption among adolescents. For example, messages that focus on catching adolescents' attention and sharing short- and long-term health consequences of high SSB consumption resonated with adolescents, but because occasional SSB intake was not seen as consequential, messages that suggest abstinence from SSBs may not be helpful in reducing consumption.

## Introduction

Approximately one in seven adolescents ages 10–17 in the United States suffers from obesity. In North Carolina, the childhood obesity rate is higher than the national average, 16.1% compared to 15.5%, respectively (1). Not only do these adolescents have health risks and complications during that developmental period, but they also have a higher likelihood of being obese adults and developing diet-related chronic disease such as type 2 diabetes, hypertension, and cardiovascular disease (2–6). They are also more likely to experience anxiety, depression, and low self-esteem, compounding the physical effects of diet-related disease (7, 8).

Sugar-sweetened beverage (SSB) intake among children is a leading contributor to obesity (9, 10) and strongly discouraged by leading child health organizations, including the Robert Wood Johnson Foundation's Healthy Eating Research Program (11). SSBs are the primary source of added sugar for adolescents and are the top contributor of empty calories in their diets (12). The National Health and Nutrition Examination Survey (NHANES) data analyses from 2003-2004 to 2013-2014 show that SSB consumption has declined in children, but these declines have been predominantly for higher-income, white children (13). SSB consumption among children is still disproportionate by racial and socioeconomic status with SSB intake higher among non-Hispanic blacks and low-income children (13, 14).

Poor dietary habits continue to be a public health problem in the United States, and parents and caregivers are the primary gatekeepers to adolescents' beverage consumption (15). Low-income adolescents who are most at risk for poor diets are often eligible for the Supplemental Nutrition Assistance Program Education (SNAP-Ed), the nutrition education component of the Supplemental Nutrition Assistance Program (SNAP) (16). The goal of SNAP-Ed is to improve the likelihood that persons eligible for the Supplemental Nutrition Assistance Program (SNAP) will make healthy food and lifestyle choices that prevent obesity (16).

North Carolina State University's (NCSU) SNAP-Ed program, Steps to Health, works to improve the diet and health of low-income North Carolinians (www.ncstepstohealth.org). Steps to Health sought to understand how North Carolina adolescents perceive and consume SSBs, and to gather data that could be used to develop a social marketing campaign that would appeal to adolescents and reduce SSB consumption. In 2020, an online survey of SNAP-eligible North Carolina adolescents ages 11–17 found that more than three-quarters of respondents (87%) reported drinking at least one SSB per day. Sodas (40%) and fruit flavored drinks (36%) were the most commonly consumed SSBs, and consumption patterns did not vary between younger (11–14 years old) and older (15–17 years old) adolescents (17). The survey also found that there was a strong association between the perceived value of SSBs and higher levels of consumption (17).

The purpose of this study was to better understand adolescents' attitudes and behaviors related to access, availability, and consumption of SSBs. A series of virtual workshops with low-income adolescents in North Carolina were conducted during the summer of 2021. In addition to general attitudes and behaviors related to SSBs, the workshops explored trusted sources of information about health and other topics, and opinions on specific types of SSB products (to gauge understanding, as well as motivators and barriers). This paper focuses specifically on the youth perceptions of SSBs.

## Materials and methods

The study authors conducted a series of virtual group workshops to explore adolescents' perceptions, attitudes, and behaviors around SSBs and SSB messaging. These workshops were designed as modifications to traditional focus groups by adjusting the location (virtual), and gathering of participants (i.e., some groups took place with each participant in a different location and on an individual screen; some participants were able to gather into a single room) to accommodate restricted protocols due to the COVID-19 pandemic.

This type of group discussion is useful to obtain detailed information about personal and group perceptions because they can provide a broad range of information and offer the opportunity to seek clarification on potentially complex or nuanced questions (18). RTI International's Institutional Review Board (IRB) designated the research and materials as “Not Human Subjects Research” and therefore exempt from review.

### Data collection

The research team's plans for the four workshop discussions were informed by research suggesting the appropriate number to suggest theme saturation in similar traditional focus groups (specifically, two to three moderated groups have been found to include at least 80% of themes; three to six groups will include 90% of themes) (19). NCSU worked with Family and Consumer Science (FCS) cooperative extension agents who deliver nutrition education for Steps to Health to recruit participants and assemble the virtual workshops. Middle and high school adolescents ages 11–17 in communities with a high proportion of SNAP eligible households were selected to participate. To ensure geographic diversity across the state, FCS agents recruited participants located in the three main regions of the state (western, central, and eastern North Carolina). Eligible adolescents were those who reported they were: (1) between the ages of 11 and 17; (2) lived in a SNAP-eligible household; (3) spoke English; and (4) had access (either individually or in a group) to a computer with the Zoom web conferencing program (20).

Before participating in the workshop discussion, parents gave their permission to have their child participate. Prior to the adolescents beginning the workshop, they provided their assent. Each workshop was conducted remotely on Zoom and was audio (but not video) recorded. To ensure privacy, a group-specific Zoom link was sent to participants or the FCS agent and a “waiting room” was enabled so that only those who the moderator admitted into the meeting were allowed to enter and participate in the workshop. To protect confidentiality, only first names were used in the discussion. Workshop discussions lasted approximately 60 min and were led by a trained moderator familiar with the research topic and a notetaker who observed and recorded detailed comments and non-verbal reactions.

To facilitate recruitment and maximize participation while considering limitations around in-person data collection and considering COVID-19 protocols, workshops were conducted using a flexible approach. This included relying on guidance from the FCS agent that recruited and assembled the groups, and the needs of adolescent participants. As a result, the workshops were conducted under two types of configurations. The first involved adolescents gathering in a single location watching the workshop facilitator and viewing stimuli on a single screen. For this configuration, the room of adolescents were not on video (the moderator could not see the youth). The FCS agent present with the adolescent participants in the room aided in facilitating the discussion. The second configuration had adolescent participants join *via* their own device (e.g., laptop or phone) with one participant per device. For this setup, adolescent participants joined from home or another location. Due to the funding source, adolescents were not compensated for participating in the group discussions.

### Workshop discussion guide development and procedures

A semi-structured discussion guide was developed containing questions related to attitudes, behaviors, norms, and consumption of SSBs, as well as items related to information sources and perceptions of SSB advertising (Appendix A). Questions were modified and expanded based on results from a 2020 online survey of SNAP-eligible North Carolina adolescents ages 11–17. The phrasing of questions had already tested with youth regarding the perceived value of SSBs and general attitudes toward them (17). This discussion guide was pilot tested with one group of adolescents to ensure that questions could be easily understood and interpreted by potential participants. No changes to the discussion guide were made before using it during the formal workshop discussions. This paper focuses on questions in the guide that centered around: (1) general health perceptions, and (2) SSB perceptions and behaviors. These sections are listed in further detail below:

#### General healthy behaviors—perceptions and behaviors

Participants were asked a series of questions to orient them to the general topic of healthy behaviors. These questions also helped ground the workshop discussion by asking what comes to mind when adolescents hear certain key phrases, including “healthy eating” or a “healthy diet.” Participants were then asked about how important “healthy eating” was (including avoiding unhealthy foods), and about who influences them when they make choices about what to eat or drink.

#### Sugar sweetened beverages—perceptions and behaviors

**Figure 1:**
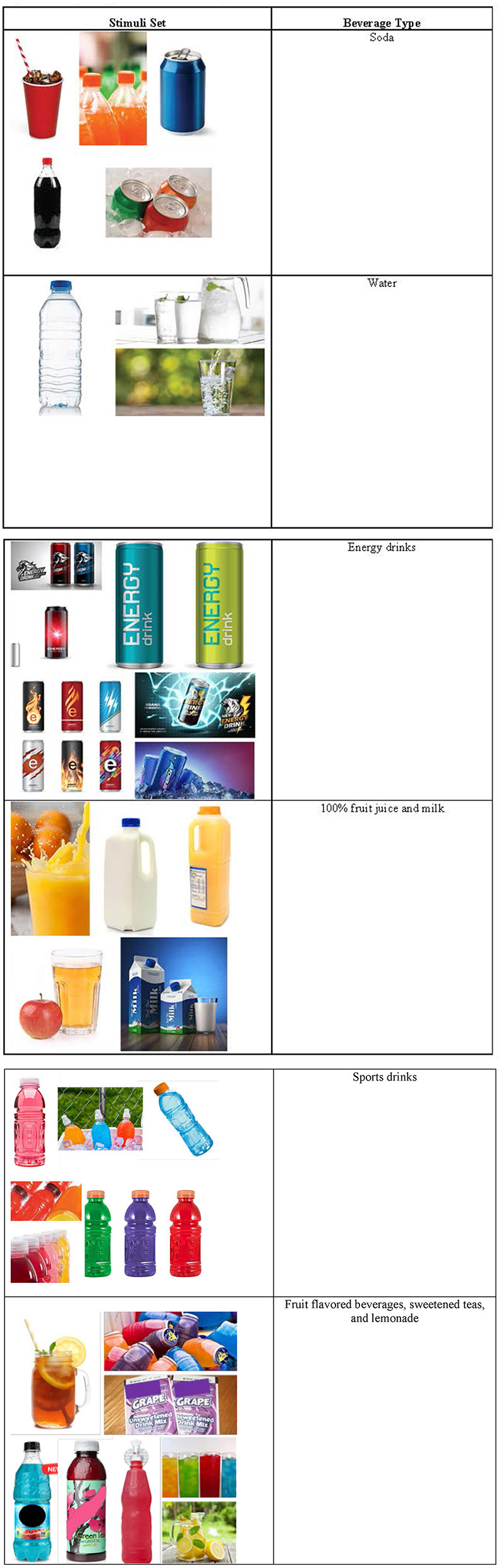
Sample sugar sweetened beverage stimuli.

Participants were shown a series of six sets of images that depicted different categories, or types, of SSBs. Each stimuli set featured a group of images representing individual products (images were generically labeled to avoid brand associations outside of the product type). Stimuli sets included: (1) soda, (2) water, (3) energy drinks, (4) 100% fruit juice and milk, (5) sports drinks, and (6) fruit flavored beverages, sweetened teas, and lemonade (see Figure 1). After seeing each set of images, participants were asked to describe their first reactions, thoughts, feelings, and opinions for each of the image sets verbally or *via* the “chat” feature in Zoom. Participants were then asked to describe their overall reaction to the phrases “sugar sweetened beverages” and “sugary drinks” and the products that the phrases represented. Adolescent participants provided details regarding situations when they chose to drink SSBs, and about parental and peer influences in those decisions. They also described health risks associated with drinking SSBs.

### Analysis

Workshop discussions were audio recorded and transcribed. Detailed notes (participant comments and non-verbal reactions, including nodding or raising hands to indicate agreement with a point), audio transcriptions, and the Zoom chat transcript were organized into a meta-matrix by moderator question. The study authors employed an inductive approach to develop a coding scheme that allowed for thematically summarizing participants' responses. Coding used the comprehensive data (notes, audio transcripts, chat records) in the matrix and were organized around knowledge, beliefs, attitudes, and perceptions around SSBs and advertising perceptions and preferences and allowed for focusing on the interpretation and meaning of the themes (21–23). The moderators (SR and KG) independently reviewed the transcripts and discussed participants' responses to questions. The two moderators (SR and KG) compared themes and reconciled any discrepancies through discussions. After discussing the participant responses, they (SR and KG) identified themes based on similar and related topics (23). Key findings are summarized below, and illustrative quotes are included to highlight participant comments to give context.

## Results

A total of four virtual workshop discussions were conducted with adolescents during the summer of 2021 (see Table 1). Two of the workshops were held in a classroom with adolescent participants viewing a large screen showing the facilitator. The other two used the format of adolescents participating from home on their individual device *via* Zoom. Approximately 36 adolescents ages 11–17 participated across the four discussions. Due to the nature of the virtual setup, during one of the workshops, facilitators were unable to see all participants who gathered in a single location through the video. The onsite FCS agent who helped facilitate reported the number of participants; however, some left early and therefore the number of participants who participated in that discussion may not be exact. Virtual workshops were held in Yadkin, Richmond, Wake, and Northampton counties in North Carolina.

**Table 1:** Workshop participants, locations, and dates.

**Group Number**	**Number of Participants**	**Location**	**Group Type**	**Date**
1	10	Yadkin county	Home with individual devices	9/07/2021
2	15*	Richmond county	Classroom with large screen	9/08/2021
3	3	Wake county	Classroom with large screen	9/14/2021
4	8	Northampton county	Home with individual devices	9/14/2021

^*^Because moderators were not able to see participants who were gathered in a single location, the number of participants who completed the Group 2 discussion may not be exact.

### Perceptions and behaviors around healthy eating or a healthy diet

When participants were asked What do you think of when you think about “healthy eating” or a “healthy diet” there was a general consensus across the adolescents that healthy eating or a healthy diet included consuming fruit, vegetables, grains, dairy, and protein. One participant mentioned healthy (lean) meat and another mentioned following *MyPlate* guidance based off the 2020 Dietary Guidelines for Americans (24). One participant commented, “*From a high school point of view, [healthy eating] is a big thing…like body image, us being healthy—a lot of girls are focusing on it*.” [Female, Group 1]. Only three participants independently mentioned beverages when asked about a healthy diet: one participant noted that a healthy diet includes drinking more water while the two other participants mentioned avoiding soda. Adolescent participants also mentioned avoiding pizza, candy, fast food, processed/pre-made food, chips, cereal, and ice-cream.

In response to the question, “Who or what influences you when it comes to making choices about what you eat or drink?” most adolescent participants stated that their parents/caregivers influenced them: “*My parents influence me the most. Friends don't have a big influence*.” [Male, Group 4]. A couple of participants mentioned friends as influential: “*[My friends] influence me because I know a lot of my drinks drink a lot of water, so I just started drinking more water sometimes*.” [Male, Group 4]. One participant noted that she sometimes ate food or beverages at her friends' houses that she would not usually consume at home: “*Some things I eat at my friends' houses are different than what I eat at home*.” [Female, Group 1].

### Perceptions and behaviors around sugar sweetened beverages

The workshop facilitator shared a series of six sets of images that depicted different categories, or types, of SSBs. Stimuli sets included: (1) soda, (2) water, (3) energy drinks, (4) 100% fruit juice and milk, (5) sports drinks, and (6) fruit flavored beverages, sweetened teas, and lemonade (see Figure 1). After seeing each set of images, participants were asked how they would “label” the type of beverage using a word or phrase (i.e., identify the category to which they belonged). There was general consensus across all workshops around the categorization of each of the SSB image sets, with participants recognizing the connection between the individual products in each set and offering similar language to label them. This was followed up by the moderator asking the participants to consider their experiences with these beverages (positive or negative opinions), situations when they might drink them, and any other opinions regarding the SSB type. We describe adolescent participant response to each of the six SSB categories below.

#### Soda image sets

When adolescents were shown the soda image set (which included soft drinks and other sugar-sweetened carbonated drinks), participants associated the images with words and phrases including “fizzy, bubbly, artificial flavors, sugar, very sweet, sticky, cold, tasty.” Adolescents shared that the soda image made them think about situations when they would drink them, such as church potlucks and cook-outs. Other participants said that the images prompted thoughts related to the immediate effects of drinking them, including feeling hyper (due to caffeine) or energized, or feeling that they fill you up so you do not each as much. One participant commented, “*[Soda] makes me think of being hyper and caffeinated because my parents always tell me if I drink too much soda that I will get hyper*.” [Male, Group 1]. In addition, several participants mentioned health risks associated with drinking soda (e.g., causing pimples, diabetes, being unhealthy): “*[Soda] tastes good, but it's not always good for you to drink all the time*.” [Female, Group 4].

When asked whether they had a positive, negative, or neutral opinion of sodas, among those participants who responded to this question, 10 participants had only positive opinions of sodas, 10 participants had only negative opinions, and 12 participants had both positive and negative opinions. Positive opinions were centered on liking the taste and thinking of special times when they drink them. Negative opinions were focused on associated health risks of consuming too much sugar. Some participants acknowledged that sodas were not good for them, but suggested they were okay to drink in moderation.

#### Water image sets

After showing adolescents the water image set (which included water, mineral water, and water with fresh fruit), participants associated the images with words and phrases including “cold, ice, refreshing, crisp, summer refresher, no sugar, healthy, beneficial”.

When adolescent participants were asked to share their initial thoughts about the water image set, they said that the images made them think about health. More specifically participants discussed how water helps keep them alive and hydrated, is beneficial for their skin (relating water consumption to acne prevention), and is necessary for the human to function: One participant made this connection by noting a perception that the body already consists mostly of water: “*You can drink [water] all the time…it doesn't hurt your body…your body is mostly water anyway so just adding water helps it*.” [Male, Group 4]. Participants also mentioned that the water image set made them think about “taste”, such as “tasty with ice” or having no sugar in it and not having any taste. When asked about situations when they would drink water, most adolescent participants mentioned water was best after playing or exercising outside when the weather is hot. Additionally, some adolescents pointed out that they could drink water all the time, as opposed to SSBs.

Among participants who responded when asked about their positive or negative opinions of water, nearly all had positive opinions of water and only two adolescents expressed some negative opinions. Positive reactions were focused on the health benefits of drinking water, water's refreshing qualities and its taste. The two participants who included negative comments suggested that some water sources (such as free tap water) may not be clean or safe to drink.

#### Energy drinks image sets

After being presented with the energy drink image set, participants associated the images with words and phrases including, “unhealthy, chemicals, espresso shots, sugary, bad for you, [brand name] energy drink, hyper, and energetic.” Of these associations, the most frequent response among adolescent participants was “hyper.” Some participants said that the images of energy drinks made them think about their wide availability. As one participant said, “*Everyone at school drinks [energy drinks] because they are in the vending machines*.” [Female, Group 1]). Additionally, a few participants mentioned examples of advertising that suggests the type of person who drinks them (specifically race car drivers). Others discussed the negative health effects from consuming too much of them: “*I heard one time that someone drank too many energy drinks and they died from that*.” [Female, Group 4].

Among participants who responded when asked about their positive or negative opinions of energy drinks, no participants had only positive opinions of energy drinks, nine participants had only negative opinions, and four participants had both positive and negative opinions. Negative attitudes focused on the associated health risks, particularly related to the impact of high levels of caffeine in the body. Those who had both positive and negative reactions acknowledged that they believed they were unhealthy, but that they liked the “boost” that the caffeine gave them, as it helped them get energy for staying engaged in school or other afterschool activities. As one participant stated, “*I know [energy drinks] are bad for you but sometimes I like to drink them when I'm tired*.” [Female, Group 1].

#### 100% fruit juice and milk image sets

When adolescents were presented with the 100% fruit juice and milk image set (which included orange juice, apple juice, and milk), adolescent participants said that words and phrases associated with this image set (participants called them “*breakfast drinks*”) included: “breakfast, fruit, healthy, and strong.” Adolescent participants said that the image of these “breakfast drinks” made them think about the taste and health related topics. When discussing taste, participants were specific: one participant commented that orange juice was too sour, and another said they didn't like the pulp in 100% orange juice. Regarding health-related topics, several participants mentioned that some people are lactose intolerant, while others stated that milk is good for their bones: “*[Fruit juice and milk] are healthy drinks and milk is good for the bones*.” [Female, Group 4]. When asked about when they would consume “breakfast drinks” participants said that they would have milk with cereal and drink the other juices mostly in the morning. As one male participant commented, “*I think [orange juice] is really good breakfast thing; you wake up and get some OJ. I eat a lot of cereal, so I like milk*.” [Male, Group 3]. Several participants simply noted that 100% fruit juice and milk were “healthy”.

Among those who responded when asked about positive and negative opinions of the image set, seven adolescents had only positive opinions, and two had both positive and negative opinions. Positive reactions included the health benefits and taste, while the negative reactions were centered on disliking the taste (mentioned above).

#### Sports drinks image sets

After showing adolescents the sports drink image set (which included sports drinks, bottles that mimicked a brand-name product, electrolyte drinks, and other energizing drinks), participants said that words and phrases associated with those beverages included, “thirst quenching, healthy, sports recovery, drinks for athletes or during physical activity, sweat, salt, and summer party drink.” When asked what they thought about sports drinks after seeing the image set, adolescent participants said they thought about specific sports or activities when they drink them (such as soccer, softball, basketball, volleyball, and football; as well “field days” at school). Some participants remembered drinking a brand name sports drink when they were sick or during the summer. Several participants noted that it could be used to replenish electrolytes and salt in the body. Lastly, specific sport drink flavors were mentioned.

Among adolescents who responded to questions about negative and positive opinions about sports drinks, four adolescents had only positive opinions and three adolescents had positive and negative opinions. Some of those who had only positive reactions cited the potential health benefits: “*[Sports drinks] helps replenish electrolytes and salt.”* [Female, Group 1]. Other participants suggested that they felt the drinks were refreshing. Those who had both positive and negative reactions liked the hydration benefits for some physical activities [as one participant noted: “*We have (sports drinks) during/after sports so you can get hydrated after your game because you might not have had as much to drink during the game.”* (Female Group 1)], but did not like the tasted or also acknowledged that they contained added sugar.

#### Fruit drinks, teas, and lemonade image sets

When adolescents were presented the fruit drinks image set (which included fruit drinks, sweetened tea, and lemonade), adolescents said they associated fruit drinks with words and phrases such as, “sweet, sugary, artificial, summer camp, unhealthy, drinks for kids, tasty, hyper, and loaded with sugar.” Adolescent participants said that the images made them think about the about taste, situations where they might consume them, health risks, and specific ingredients or brand names of fruit juices. In terms of taste, some participants felt the fruit drinks tasted “artificial” but were still “tasty” or a desirable beverage option. Places or situations where adolescent drink fruit drinks included celebrations (especially with young children) or cookouts. One participant mentioned a perceived health risk that fruit drinks can negatively impact kidney functioning. Finally, adolescent participants generally noted mentioned that sugar was a main ingredient in fruit drinks and other participants mentioned specific brands neither negatively or positively.

Among adolescent participants who responded to the question about positive and negative opinions about fruit drinks, a few had both positive and negative opinions. Those who had negative opinions cited the health risks associated with consuming too much sugar: “*They are not very healthy and loaded with lots of sugar*.” [Female, Group 4]. Those with positive reactions noted, “*[Fruit drinks] are nostalgic but unhealthy*” [Female, Group 1] when talking about them in relation to past celebrations or other social gatherings.

### Conclusions

This study provides several key insights regarding adolescents' perceptions, attitudes, and consumption behaviors around SSBs in NC. First, research on the correlation between low-income adolescents and health literacy is mixed. This study shows that low-income adolescents participating in our workshop discussions had fairly high health literacy regarding the harms of consuming SSBs. For the purposes of this discussion, health literacy “is the extent to which individuals attain, manage, and understand health information and apply that information in health decision-making” (25). In a 2018 systematic review of adolescent health literacy and health behaviors (26), among five studies that examined the relationship between income and health literacy, four studies found that having lower incomes was associated with lower health literacy, while only one found no statistically significant relationship between the two (27). However, since adolescents in this study were recruited from SNAP-Ed classes, this may have increased their health literacy as the curriculum focuses on promoting the consumption of beverages low in added sugar, and the negative health impacts of consuming large amounts of SSBs.

Additionally, adolescent responses revealed that they viewed their parents/caregivers as role models in terms of what beverages to drink. Other research supports that children and adolescents look to their parents/caregivers for guidance and often mirror or mimic their health behaviors (28). This is consistent with a 2012 study that showed parent support for healthy beverage consumption was associated with reduced SSB consumption among 541 children between the ages of 5 and 8 years old (28). Additionally, since parents/caregivers are more likely to be responsible for stocking foods and beverages at home, if they purchase SSBs, children are more likely to consume them (29). Therefore, raising parents' and caregivers' awareness of the impacts that their own health behaviors have on their children continues to be a promising public health strategy to curb SSB consumption among adolescents.

Strategies for raising awareness among parents and caregivers can take many forms, including through social marketing—the use of consumer marketing techniques (e.g., audience segmentation, advertising campaigns) to promote voluntary behavior change to achieve positive population-level effects (30). Findings from this study will be useful in informing the development of a social marketing campaign aimed at reducing SSB consumption among adolescents. For example, messages that focus on catching adolescents' attention and sharing both short- and long-term health consequences of high SSB consumption may resonate with adolescents. However, because occasional SSB intake was not seen as consequential among workshop participants (e.g., consuming soda or sweet tea during special occasions), messages that suggest abstinence from SSBs may not be helpful in reducing consumption.

Previous research on social marketing campaigns and interventions implemented in the United States and Europe have targeted adolescent SSB consumption with positive effects (31–33). For example, the evaluation of a campaign in the Netherlands that promoted the consumption of water over SSBs directly to adolescents found that the intervention was related to an overall reduction in SSB consumption (31). Communication campaigns targeting the influence of parents have had similar results (32, 33). For example, exposure to a city-wide media campaign in Philadelphia that targeted parents with a child between the ages of 3–16 years to reduce SSB consumption was significantly associated with the parents' intent to substitute non–sugary drinks for SSBs for their children (33). However, there are still gaps in our understanding of how adolescents understand and receive SSB-specific social marketing campaign messages and materials, particularly when media messages are intended to reach them directly.

Like all research, this study had several limitations. Due to protocols for research during the COVID-19 pandemic, the workshops were virtual, which required a hybrid set-up of all adolescents watching one large screen or adolescents being on their individual screens. Some adolescents did not have their cameras enabled, so it was difficult to gauge some non-verbal responses or cues to the moderator's questions. Because of this adaptation, the workshops were not traditional focus groups; however, the consistent application of a single discussion guide, stimuli presentation, and coding scheme allowed us to summarize findings across all group discussions. The virtual environment may have also had some advantages over in person group research, including facilitating more participant diversity by reducing some barriers (e.g., transportation, time) and encouraging contributions to the discussion, and has been used successfully to collect information with variety of audiences, including adolescents (34).

Additionally, due to the small sample size and restrictive geographic location (adolescents had to live in NC), results may not be generalizable to adolescent populations in other states. Lastly, since adolescents were already enrolled in SNAP-Education classes, they may have had higher health literacy regarding the harms of SSBs, which could have influenced their responses in the discussion. In addition, as a qualitative study with a self-selected group of participants, there are limitations in the generalizability of findings.

Each group included a facilitator who led the discussion and a dedicated notetaker, who captured detailed notes including verbal comments and other observations about the group. Group discussions were also audio recorded and transcribed. The combination of these data allowed for a comprehensive review of participants' responses and reactions. The use of thematic analysis based off the combination of detailed notes that captured non-verbal responses (e.g., raised hands, nodding, and other indicators of agreement where possible), audio transcriptions and chat transcripts from each of the workshops is a strength in that it focuses on the interpretation and meaning of themes (22). Lastly, both of the workshop facilitators independently reviewed the data and coded responses. Discrepancies around interpretation of themes were discussed until agreement was reached. During this process, they identified themes based on similar and related topics to reach a consensus.

This study reveals several important themes, including that adolescents have both positive and negative opinions regarding six different types of beverages: (1) soda, (2) water, (3) energy drinks, (4) 100% fruit juice and milk, (5) sports drinks, and (6) fruit flavored beverages, sweetened teas, and lemonade. The information we have learned about adolescent perceptions of these drinks could help contribute to the development of messages aimed at reducing SSB consumption. Future research should continue to examine adolescent perceptions, attitudes, and consumption behaviors around SSBs. Additionally, raising awareness among parents/caregivers regarding the level of influence on their children is an important factor to consider. SSB intake among adolescents is a leading contributor to obesity and other diet-related chronic diseases. Researchers and public health practitioners should continue to examine strategies and interventions aimed at decreasing SSB consumption.

## Data availability statement

The raw data supporting the conclusions of this article will be made available by the authors, without undue reservation.

## Ethics statement

The studies involving human participants were reviewed and approved by RTI International's Institutional Review Board. Written informed consent to participate in this study was provided by the participants' legal guardian/next of kin.

## Author contributions

LH-M, SR, and KG: conceptualization and methodology, writing—review and editing, and writing—original draft preparation. SR and KG: analysis and investigation. LH-M: funding acquisition. All authors have read and agreed to the published version of the manuscript.

## Funding

The study was funded by the United States Department of Agriculture's Supplemental Nutrition Assistance Program-Education (SNAP-Ed) (Grant #00041221).

## Conflict of interest

The authors declare that the research was conducted in the absence of any commercial or financial relationships that could be construed as a potential conflict of interest.

## Publisher's note

All claims expressed in this article are solely those of the authors and do not necessarily represent those of their affiliated organizations, or those of the publisher, the editors and the reviewers. Any product that may be evaluated in this article, or claim that may be made by its manufacturer, is not guaranteed or endorsed by the publisher.
