# Supplementary material for: Perceptions of sugar-sweetened beverages among adolescents in North Carolina
Source: Front Public Health. 2022 Sep 29;10:943295. doi: 10.3389/fpubh.2022.943295 (PMC9557148; doi:10.3389/fpubh.2022.943295)
Supplement: Supplementary file 1 [file Data_Sheet_1.pdf]

Appendix A: Focus Group Moderator Guide

1. General Healthy Behaviors – Perceptions and Behaviors	
I'd like to start the discussion by getting your general thoughts about what you consider to be healthy behaviors.	
What do you think of when you think about "healthy eating" or a "healthy diet"?	PROBE:<br>• How do you think a "balanced" diet is part of healthy eating? What does a "balanced" diet mean to you?<br>• How important is avoiding some kinds of foods or drinks (for example, sugary snacks)?
Who or what influences you when it comes to making choices about what you eat or drink?	PROBE:<br>• How much influence do your parents (or caregivers) have?<br>• How much influence would you say that your friends have?

2. Sugar Sweetened Beverages – Perceptions and Behaviors	
Now I'd like to shift to talk specifically about *beverages,* or the things that you *drink.* First, we are going to show you a series of pictures of different beverages, or drinks.<br><br>This is a "top of mind" activity, so we'd like you to think of the first few words or phrases that come to mind when you see these images.<br><br>**FIRST,** please take a look at the images and think about the overall beverage *types or categories* represented. There is a place to "label" it at the top (printed stimuli)/enter it in the chat.<br><br>Remember, we are wondering how you would label the *type* of beverage (using a word or phrase). We have removed brand names, so we aren't as interested in guessing or identifying which brands they represent.<br><br>**NEXT,** in the second column/for the second question, we would like for you to think about the images overall, and you should consider your experiences with these beverages, situations when you might drink them, your opinions about them, and anything else that comes to mind.<br><br>We will then discuss your responses as a group.	
[1ST IMAGE – SHOW ON SCREEN]:<br><br>[MODERATOR ASK PARTICIPANTS TO SHARE INITIAL THOUGHTS]<br><br>[REPEAT FOR ALL IMAGE SETS]	PROBE:<br>• Would you say that you have a positive, negative, or neutral opinion of these beverages? Why?<br>• What kinds of things did you think about when you saw these images? For example, did you think about your experiences or situations when you want to or have to drink them?
